# Effects of topical fluoride treatment on the bond strength of pit and fissure sealants: A systematic review

**DOI:** 10.34172/joddd.2023.39160

**Published:** 2023-07-17

**Authors:** Naimeh Teimoory, Katayoun Katebi, Armin Ghahramanzadeh, Ali Vafaei

**Affiliations:** ^1^Department of Pediatric Dentistry, Faculty of Dentistry, Tabriz University of Medical Sciences, Tabriz, Iran; ^2^Department of Oral and Maxillofacial Medicine, Faculty of Dentistry, Tabriz University of Medical Sciences, Tabriz, Iran; ^3^Department of Pediatrics, Faculty of Medicine, Bam University of Medical Sciences, Bam, Iran

**Keywords:** Bond strength, Fluoride therapy, Pit and fissure sealant

## Abstract

**Background.:**

This systematic review aimed to evaluate the available scientific evidence concerning the effects of topical fluoride treatment on the bond strength of pit and fissure sealants. Prevention of dental caries is one of the crucial issues in pediatric dentistry. Pit and fissure sealant and fluoride therapies are two caries prevention procedures that may be performed in one session. However, fluoride therapy may affect the bond strength of pit and fissure sealants.

**Methods.:**

An electronic search for in vitro studies published in English and Persian on topical fluoride therapy and the bond strength of pit and fissure sealants was performed via PubMed/ Medline, Web of Science, Google Scholar, Embase, and Scopus databases until May 2022. The articles were independently reviewed for quality by two reviewers. Textual data were analyzed manually, and the bond strength of sealants placed after fluoride application was compared with control groups.

**Results.:**

A total of 8482 articles were initially identified and reviewed by two independent reviewers, and 13 were selected for full-text evaluation. Finally, six articles were included in the systematic review. A total of 250 teeth were studied, 148 of which were in the case group (fluoride group) and 102 in the control group. Tensile and shear bond strengths were compared between groups in the studies.

**Conclusion.:**

In the studies in which the tooth surfaces were washed after applying fluoride, there was no change in the fissure sealant bond strength. However, in studies in which fluoride was not washed, the bond strength decreased significantly, independent of the fluoride type.

## Introduction

 Dental caries is the most common chronic disease in childhood.^1^ More than 50% of 5–9-year-old children and 78% of 17-year-olds have at least one decayed or filled tooth.^2^ Dental pit and fissure sealants and topical fluoride therapy are two essential strategies for caries prevention.^3^ Pit and fissure sealants provide a physical barrier against microorganisms and nutrients. This barrier prevents the initiation of dental caries and helps arrest the existing incipient caries.^4^ It has been shown that permanent molars which have received occlusal sealants remain caries-free for up to 48 months compared to molars that are not sealed.^5^ The caries-preventive efficacy of dental sealants depends on their retention. Many factors can contribute to sealants’ failure, including application technique errors and physical and chemical characteristics of the sealant materials, such as viscosity and light-curing conditions. Furthermore, inadequate etching can result in sealant failure.^6-9^

 One factor that affects pit and fissure treatment effectiveness is the use of topical fluoride before sealant placement. The surface of filled sealants has been reported to weaken when topical fluoride gels are used before dental sealants application.^10^ Historically, fluoride use has been contraindicated before placing pit and fissure sealants since it has been proposed that fluoride reduces enamel solubility in acid, preventing complete etching of the enamel surface.^11,12^

 Fluoride therapy is one of the most common and beneficial preventive procedures for children’s teeth. Fluoride varnishes have been reported to be effective in caries prevention of permanent teeth when applied at 3- or 6-month periods after the first tooth erupts.^13^ Fluoride varnish contains 50 000 ppm sodium fluoride in a resin varnish. After its application, it adheres to the surfaces of the teeth for several hours.^14^ Then, the fluoride reservoirs within plaque and teeth absorb the fluoride ions.^14^ Since different types of fluoride are available, the ADA clinical guidelines recommend 2.26% fluoride varnish or 1.23% acidulated phosphate fluoride (APF) gel for individuals at risk of dental caries, who are at least 5 years old.^15^ The low pH of 1.23% APF results in the dissolution of the enamel surface to form calcium fluoride (CaF_2_).^16^ APF has been shown to decrease the surface roughness on the enamel of both primary and permanent teeth,^17,18^ which can affect the adhesion of resins such as composite resins and fissure sealant materials to the enamel.

 Studies have reported conflicting results regarding the use of fluorides before sealants. Some studies have reported that fluoride interferes with bonding pit and fissure sealants to enamel, while others have reported that fluoride can improve bonding. Low et al reported that 8% stannous fluoride applied before tooth etching increased the tensile bond strength.^19^ However, a recent study suggested that APF does not negatively affect the bond strength of composite resins or sealants to enamel.^20^

 Therefore, this study aimed to systematically review the available literature regarding the application of fluoride before pit and fissure sealant placement since this synchrony can reduce children’s dental office visits.

## Methods

 In this systematic review, the principal study question was formulated based on the “PICO (population, intervention, comparison, and outcome)” approach in which “P” was teeth that were to receive fissure sealant treatment, “I” was topical fluoride treatment, “C” was topical artificial saliva, and “O” was the change in bond strength of pit and fissure sealants. This study aimed to answer, “Does topical fluoride treatment decrease the bond strength of pit and fissure sealants compared to artificial saliva?”

 Inclusion criteria were in vitro studies, studies with non-carious teeth, studies using topical fluoride, studies using fluoride before etching, articles in English and Persian, and papers published until May 2022. Exclusion criteria were in vivo studies and letters to editors, studies using fluoride after etching, reprint articles using the same sample data, and non-human research.

###  Databases and search strategy 

**Figure 1:**
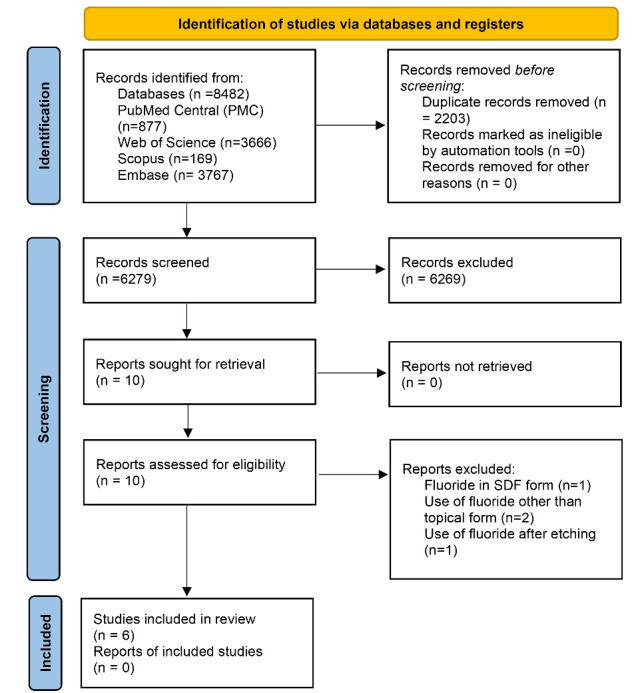
The PRISMA flowchart of the selection process of the systematic review

 The article selection processes were performed in four steps conforming to the PRISMA flow diagram (Figure 1).^21^ The electronic search was conducted in PubMed/Medline, Web of Science, Google Scholar, Embase, and Scopus databases. The key words were selected based on Medical Subject Heading (MESH) terms and free terms. The search keywords were:

 “fissure sealant,” “sealant,” “pit and fissure sealant,” “sealant therapy,” “fissure sealant therapy,” “pit and fissure sealant therapy,” “fluoride,” “fluoride therapy,” “fluoride treatment,” “topical fluoride,” “sodium fluoride,” “NaF,” “stannous fluoride,” “SnF,” “SnF2,” “Snf2,” “acidulated phosphate fluoride,” “APF,” “fluoride gel,” “fluoride varnish,” “topical fluoride therapy,” “topical fluoride treatment,” “bond strength,” tensile bond strength,” “shear bond strength,” “tension bond strength,” “retention,” “debonding,” and “failure.”

 Every possible combination of free and MESH terms with the “OR” and “AND” operators was considered for finding data. In addition, the research team tried to communicate with the corresponding authors for supplementary information if necessary. The reference lists of selected studies were also searched to identify more research.

 The EndNote Basic software was used to manage the references, and duplicate references were identified and removed.

###  Study selection 

 Two independent reviewers (KK and NT) scanned the titles and abstracts of the articles. In the next step, the full texts of the selected articles were reviewed. In the case of a disagreement between the two reviewers, a third reviewer (AG) was consulted. Finally, data from the included articles were extracted using a pre-designed data extraction sheet. A customized form for data extraction was built by Microsoft Excel software to classify the details of the studies, like study ID (first author’s name and year of publication), sample type, groups, sample size, type of bond strength test, crosshead speed, bond strength in MPa.

###  Assessment of the risk of bias

 The Cochrane risk-of-bias tool for randomized trials version 2 (RoB2)^22^ was utilized by two independent reviewers (KK and NT) to appraise the selected articles, thus assessing the risk of bias in studies. Disputes were resolved by a discussion with a third reviewer (AG). Studies with a high risk of bias, including studies without a control group and studies in which the randomization was not specified, were excluded.

## Results

 Of 8482 articles initially identified, 6279 studies remained after the duplicates were removed. After reading the titles and abstracts, 6266 articles were excluded, and the remaining 10 full-text articles were dependently reviewed by two investigators. According to the predefined inclusion and exclusion criteria, one article was excluded because it used fluoride in silver diamine fluoride (SDF) form^23^ since its composition might have affected the results. Also, two articles used fluoride other than in topical form.^24,25^ Seven articles examined the effect of fluoride in topical form, of which one used fluoride after etching^26^ and six used etching after fluoride therapy.

###  Evaluation of the risk of bias results

**Figure 2:**
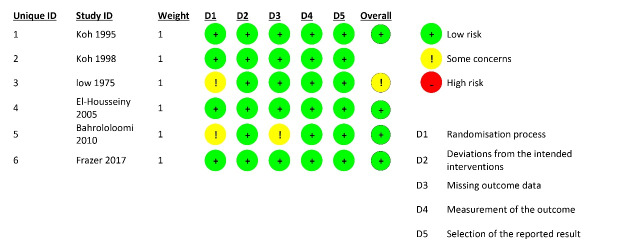
The results of the assessment of the risk of bias

 None of the studies showed a high risk of bias. According to the Cochrane risk-of-bias tool for randomized trials version 2 (ROB2),^22^ five articles showed a low,^10,27-30^ and one showed a moderate risk of bias.^19^ The details are presented in Figure 2.

###  Characteristics of studies

 The descriptive characteristics and the associated data of the included studies are presented in Table 1. A total of 250 teeth were studied, 148 of which were in the case group (fluoride group), with 102 in the control group. All the studies used the buccal surfaces of the teeth, except for one study by Frazer et al,^30^ which used the lingual surfaces of mandibular molars and buccal surfaces of maxillary molars. All the studies used molar teeth except for a study by Bahrololoomi et al, which used premolar teeth.^29^ Koh et al,^27^ Koh et al,^28^ and Low et al^19^ evaluated tensile bond strength, while El-Housseiny and Sharaf,^10^ Bahrololoomi et al,^29^ and Frazer et al^30^ evaluated shear bond strength.

**Table 1:** The descriptive characteristics and the associated data of the included studies

**Article ID**	**Sample type**	**Groups**	**Sample size per group**	**Type of sealant**	**Type of bond strength test**	**Crosshead speed**	**Bond strength (MPa)**	**Risk of bias**
Koh et al, 1995 ^27^	The facial surface of non-carious permanent molar teeth	Artificial saliva	5	Unfilled (concise, Lot#4TE, 3M)	Tensile bond strength	0.05 cm/min	20	Low risk
		1% Topical NaF					26	
		1.64% Topical SnF					15	
		1.23% Topical APF					21	
		Artificial saliva		Filled (lot#089064, Bisco, Itasca, IL)			30	
		1% Topical NaF					27	
		1.64% Topical SnF					25	
		1.23% Topical APF					21	
Koh et al, 1998 ^28^	The facial surface of non-carious permanent molar teeth	Artificial saliva	5	Unfilled (concise, Lot no 3PU, 3M)	Tensile bond strength	0.05 cm/min	15 ± 4	Low risk
		1% Topical NaF					21 ± 4	
		1.64% Topical SnF					14 ± 3	
		1.23% Topical APF					18 ± 4	
		Artificial saliva		Filled (lot No. 960402, Caulk)			24 ± 5	
		1% Topical NaF					19 ± 4	
		1.64% Topical SnF					22 ± 6	
		1.23% Topical APF					18 ± 5	
Low et al, 1975 ^19^	Not reported	Control (nothing was applied)	Not reported^*^	Polymeric Nuva seal	Tensile bond strength	0.5 cm/min	36.6 ± 9.0	Moderate risk
		1.23% Topical APF	16				4.9 ± 3.3	
El-Housseiny, 2005 ^10^	The buccal surfaces of sound second permanent molar teeth	Control (nothing was applied	10	Vesioseal, ESPE	Shear bond strength	5 mm/min	70.9 ± 55.0	Low risk
		1.23% topical APF		Vesioseal, ESPE			64.8 ± 51.4	
Bahrololoomi et al, 2010 ^29^	The facial surfaces of non-carious permanent premolar teeth	Control (nothing was applied	14	F Seal Helio	Shear bond strength	1 mm/min	17.7 ± 5.2	Low risk
		1.23% topical APF		F Seal Helio			19.6 ± 4.2	
Frazer et al, 2017 ^30^	The lingual sur faces of mandibular molars and buccal surfaces of maxillary molars of non-carious permanent molar teeth	Control (nothing was applied	48	3M ESPE, St. Paul, Minn., USA	Shear bond strength	1 mm/min	15.5 ± 6.0	Low risk
		5% topical NaF varnish		3M ESPE, St. Paul, Minn., USA			0.4 ± 0.3	

APF, acidulated phosphate fluoride; MPa, Megapascal. *The criterion for comparing two parameters in this study (between the case and control groups) was a previous article by the researcher as mentioned in the discussion section.^31^

## Discussion

 Dental pit and fissure sealants and topical fluoride therapy are two essential strategies for caries prevention that may sometimes be accomplished in the same visit.^2^ However, there are some concerns regarding the negative effect of fluoride treatment that might influence the bond strength of fissure sealants. Therefore, we designed this review to evaluate this issue.

 In Koh et al^27,28^ studies, the teeth were first treated using one topical fluoride method. Then all the samples were rinsed. The step was followed by etching and placing a fissure sealant. Finally, the tensile bond strength was measured. According to the results, topical fluoride treatment by either sodium fluoride, stannous fluoride, or acid phosphate-fluoride had no significant clinical effect on the retention of pit and fissure sealants.

 In Low and colleagues’ study,^19^ the samples were first treated with an acid phosphate-fluoride solution for 4 minutes. Next, the surfaces were dried with gauze to remove the excess fluoride solution. Then the samples were etched, and the sealant was applied. Finally, the tensile bond strength was measured. According to the results of tensile bond strength tests, using APF as the topical fluoride agent is contraindicated after the combined application of topical stannous fluoride and fissure sealant with Nuva Seal.

 El-Housseiny and Sharaf^10^ and Bahrololoomi et al^29^ used pumice on samples, then rinsed and dried them. Then, APF gel was applied on surfaces, left for 4 minutes, and rinsed and dried. Then all the samples were rinsed. The steps were followed by etching and placing the fissure sealant. Finally, they measured the shear bond strength. They found that applying fluoride before etching did not adversely affect sealant bonding. El-Housseiny and Sharaf^10^ also mentioned that “there are clinical and practical advantages to placing sealant immediately after topical fluoride application. First, if the sealant is lost, the tooth structure underneath it will benefit from fluoride; compared to a newly erupted tooth that was sealed without exposure to fluoride treatment, the former would be more resistant to caries. Second, patients who have received a fluoride treatment would not need to be rescheduled later for sealant placement.”

 Frazer et al^30^ applied a thin 5% sodium fluoride (NaF) varnish with a dry microbrush to the enamel surfaces. The teeth were etched five minutes later, and dental sealant was placed. Finally, they measured the shear bond strength. They showed that fluoride varnish applied immediately before pit and fissure sealant placement negatively affected the shear bond strength of the sealant.

 In all the studies, the tooth surface was washed after applying fluoride, except for two studies by Low et al and Frazer et al.^19,30^ This might be the reason for differences in the results of fissure sealant bond strength tests. However, in routine clinical practice, it is recommended to let the fluoride remain on the tooth surface for at least 30 minutes, and the tooth should not contact water.^32^ This decrease in bond strength in these two studies might not be related to changes in the enamel structure due to fluoride therapy but to excess material on the grooves due to not washing the excess fluoride and simply removing the excess amount by gas.

 Our search had some limitations in finding relevant articles; the full texts of three articles could not be found, and we could only check Persian and English articles. Also, some differences in these papers made us unable to perform a meta-analysis, such as differences in crosshead speeds and bond strength types measured. Additionally, this systematic review mostly included small trials with limited samples, which might have affected the results.

## Conclusion

 In studies where the tooth surfaces were washed after using fluoride, followed by using an acid etchant, there was no change in fissure sealant bond strength. However, in studies in which fluoride was not washed, followed by using an acid etchant, the bond strength decreased significantly, independent of the fluoride type. Since washing the fluoride is not recommended in clinical practice, it is better to postpone the fissure sealant therapy for another session.

## Acknowledgments

 The authors thank Mr. Hossein Hosseinifard for the statistical consult.

## Competing Interests

 The authors declare no conflict of interest.

## Ethical Approval

 Not Applicable.

## Funding

 None.
